# Supplementary material for: Diverse secondary metabolites are expressed in particle-associated and free-living microorganisms of the permanently anoxic Cariaco Basin
Source: Nat Commun. 2023 Feb 6;14:656. doi: 10.1038/s41467-023-36026-w (PMC9902471; doi:10.1038/s41467-023-36026-w)
Supplement: Supplementary file 1 — Supplementary Information [file 41467_2023_36026_MOESM1_ESM.pdf]

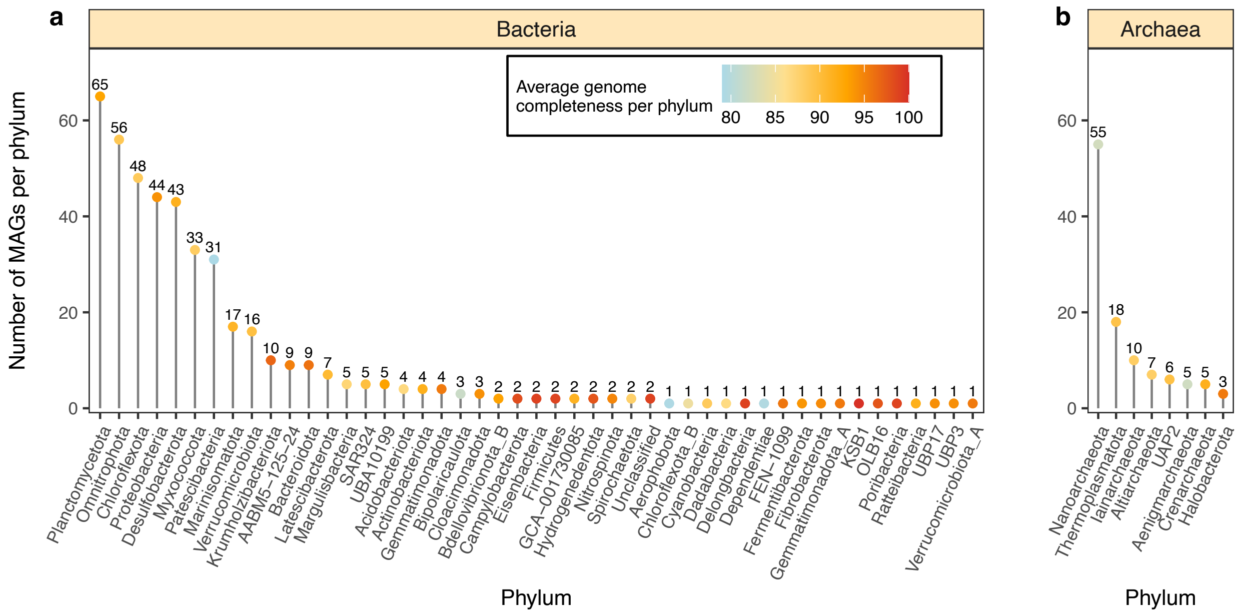

Supplementary Figure 1: Frequency of Cariaco prokaryotic MAGs (≥75% completeness, ≤5% contamination) by bacterial (a) and archaeal phylum (b). Colored dots at the end of each line segment correspond to the mean genome completeness of the phylum; the number above the dot quantifies the number of genomes recovered from the phylum. Source data are provided as a Source Data file.

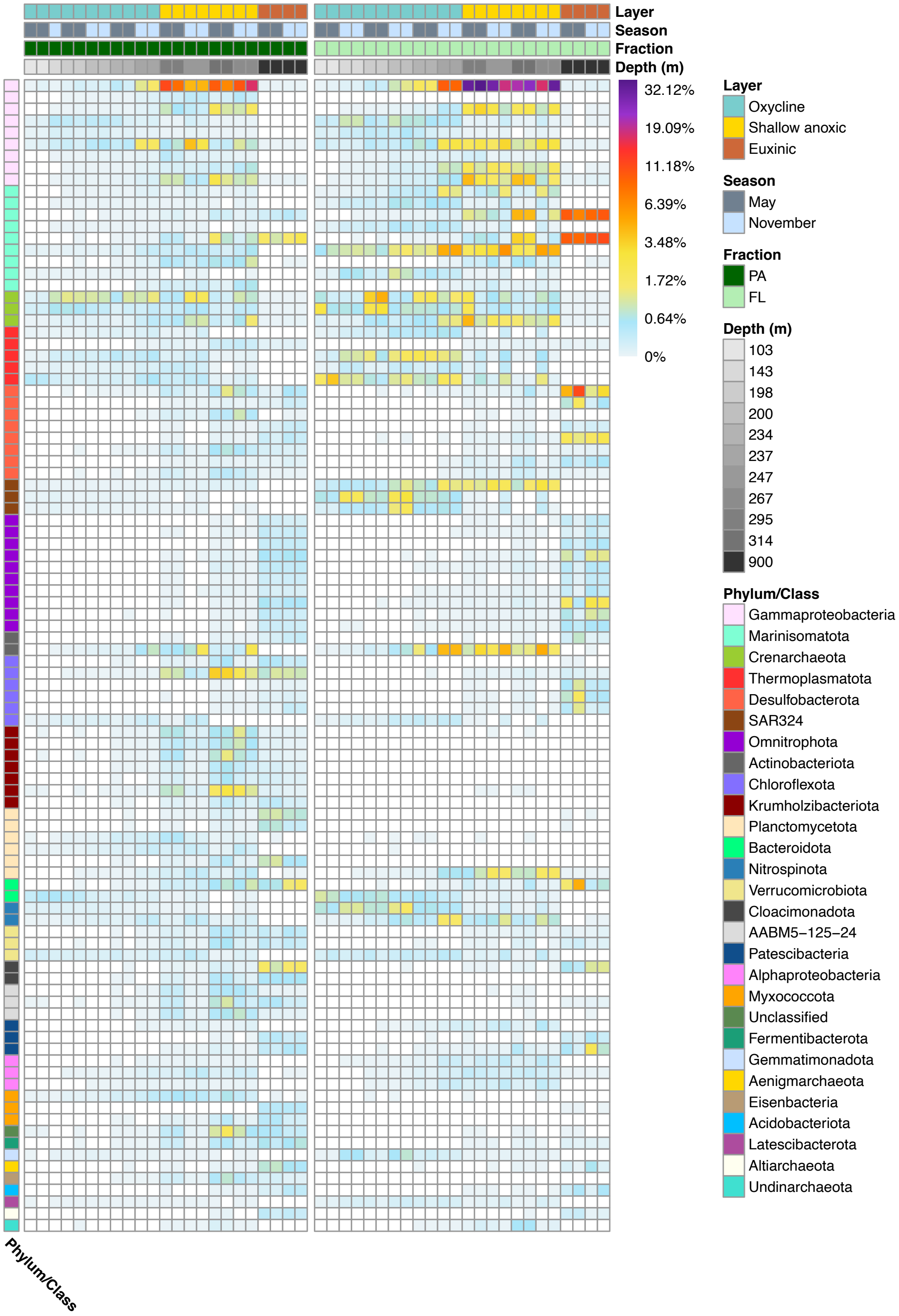

Supplementary Figure 2: MAG relative abundances. The heatmap shows for each column the percentage of total pre-processed reads from a metagenomic sample that mapped to the 100 most abundant MAGs (across all samples) using a log1p scale ranging from 0-32.12% relative abundance. Each row represents an individual MAG (additional information about the MAGs can be found in Supplementary Tables 1-2, 4 and 5). Source data are provided as a Source Data file.

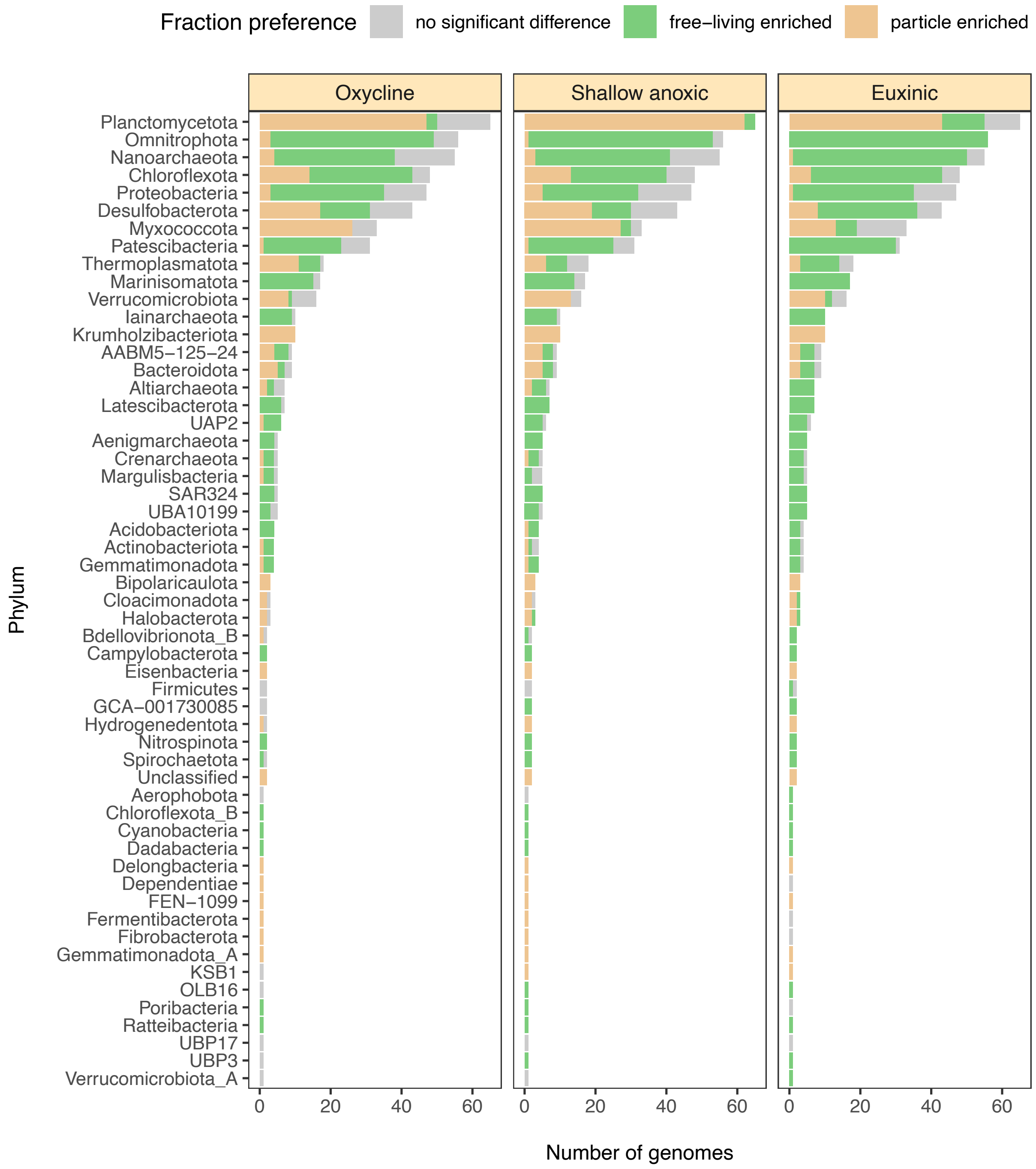

Supplementary Figure 3: MAG differential abundance (fraction preference). Bar charts of the differential abundances of MAGs, grouped by phylum (DESeq2; $P < 0.05$; False Discovery Rate (FDR) = 5%) for each layer of the water column (oxycline, shallow anoxic, euxinic). The light brown color indicates MAGs that were more abundant in the PA metagenomes, the green color represents MAGs that were more abundant in the FL metagenomes, and the grey color indicates there was not statistically significant difference between the FL and PA fractions. Phyla represented in all three panels are from groups for which at least 5 genomes were recovered. Source data are provided as a Source Data file.

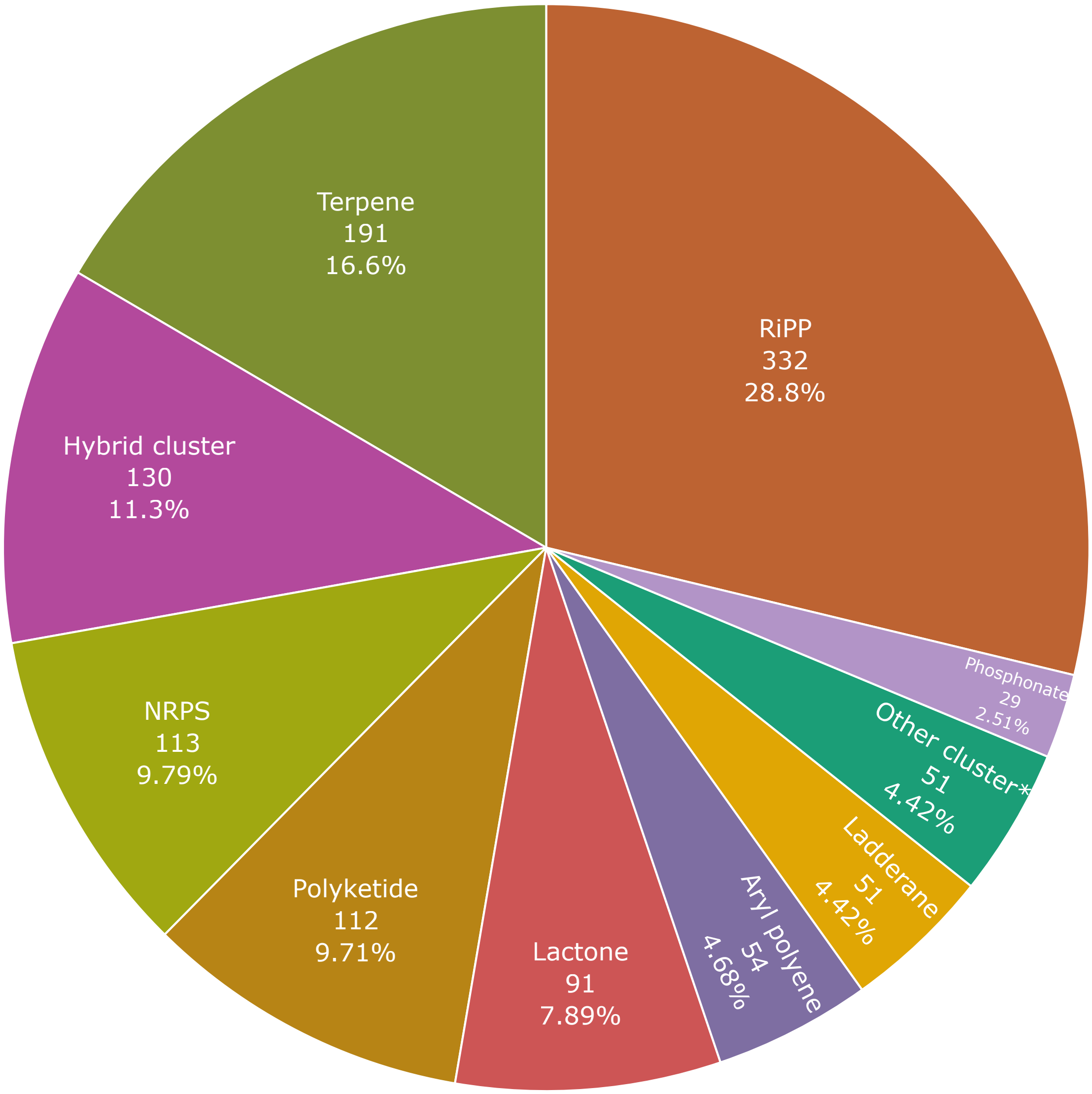

Supplementary Figure 4: Distribution of the biosynthetic gene clusters identified using antiSMASH 6.0. Each label corresponds to the total amount of biosynthetic gene clusters >= 10kb recovered from a given class, with the total number listed below the class followed by the percentage (%) out of the total BGC count (1,154). Other cluster* includes resorcinol, nucleoside, linear azol(in)e-containing peptide, acyl amino acid, cyclodipeptide, ectoine, redox-cofactor, non-alpha poly-amino group acid, siderophore, polybrominated diphenyl ether, and indole biosynthetic gene clusters. Source data are provided as a Source Data file.

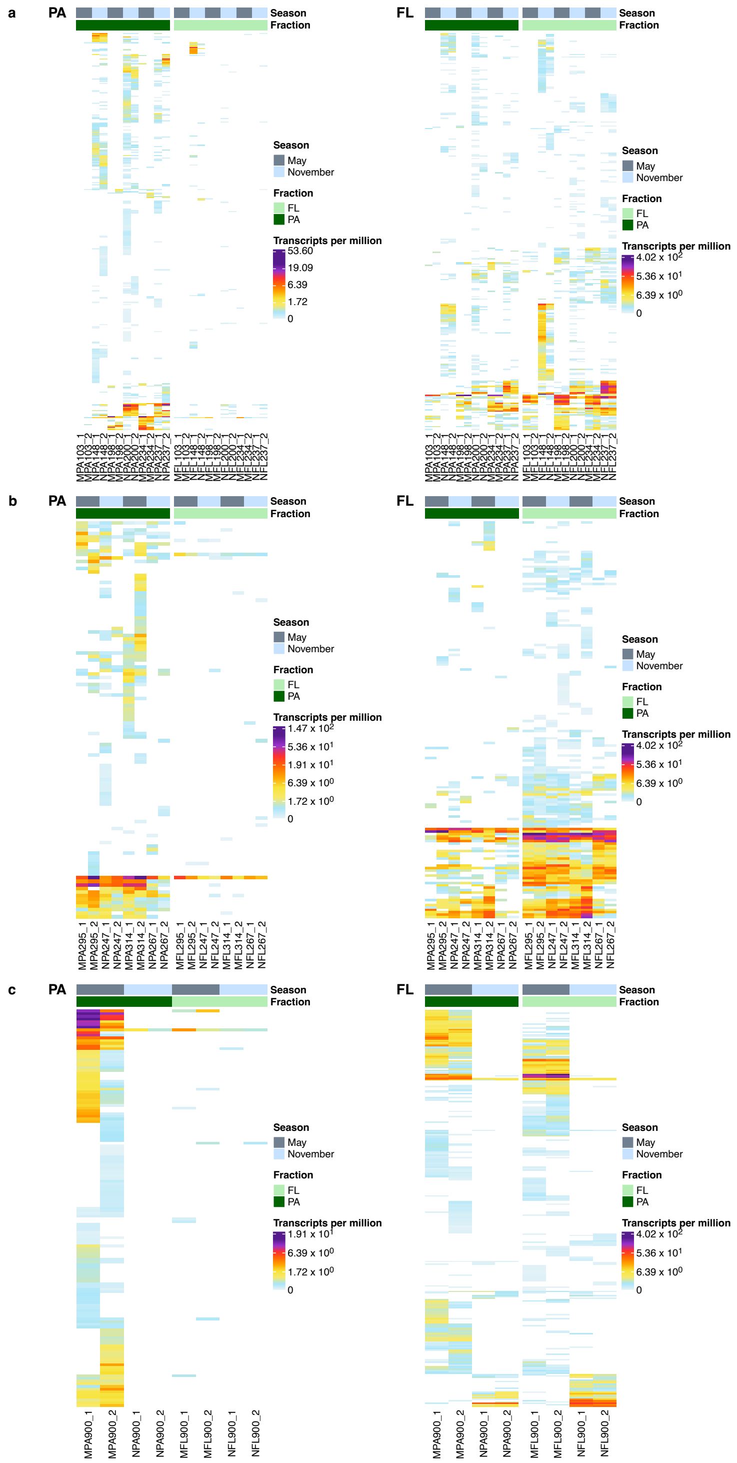

Supplementary Figure 5: Biosynthetic transcript expression of MAGs with strict PA and FL fraction preferences. Heatmaps of the expression (transcripts per million) of biosynthetic gene cluster transcripts from MAGs with an apparent PA fraction preference (DESeq2; $P < 0.05$; False Discovery Rate (FDR) = 5%) in left-hand panels labelled "PA", and from MAGs more abundant in the FL fraction in the right-hand panels labelled "FL" from **a** oxycline, **b** shallow anoxic and **c** euxinic water layers. Source data are provided as a Source Data file.

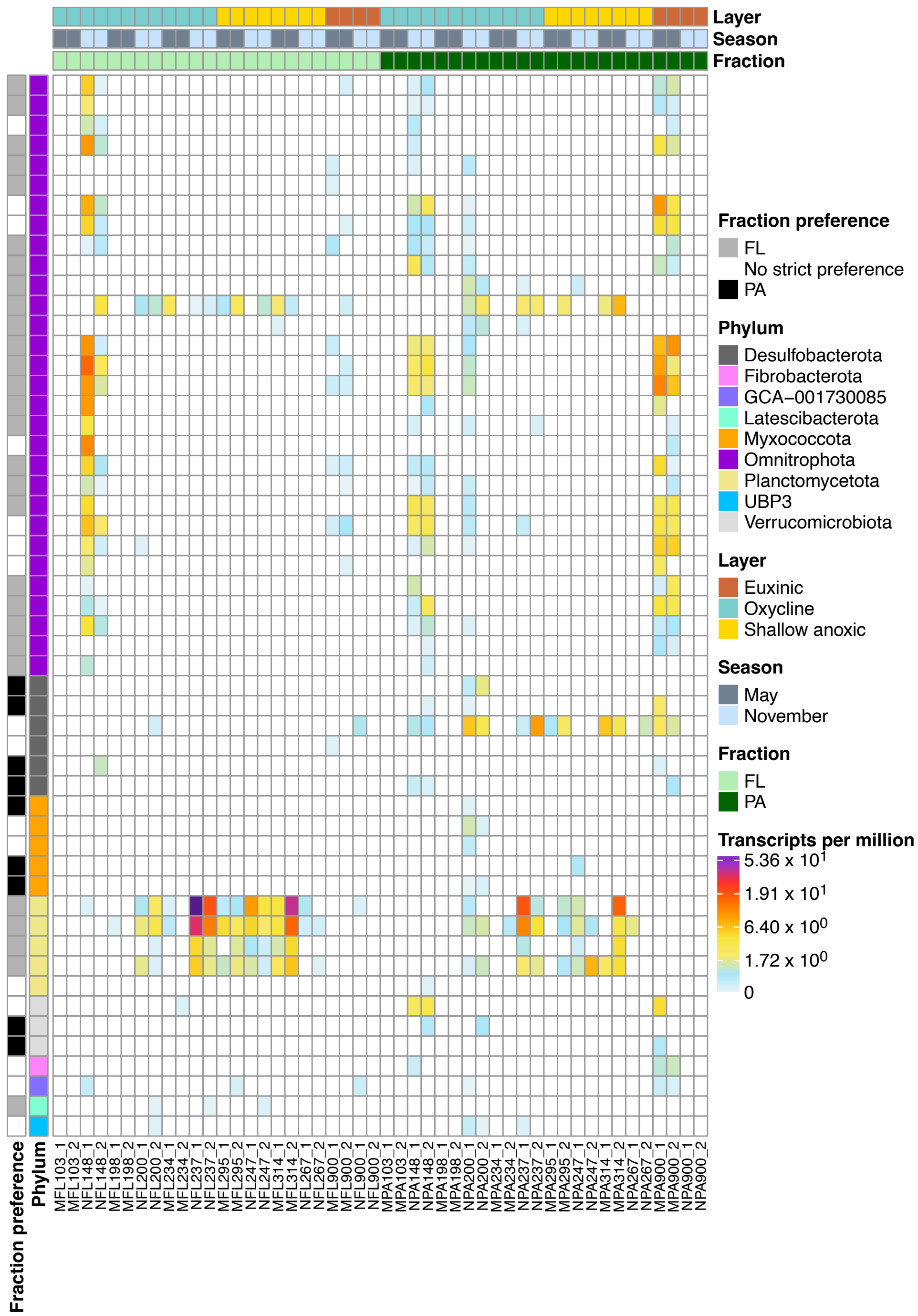

Supplementary Figure 6: Expression of biosynthetic transcripts from gene clusters annotated as ladderanes. Heat map of the expression (transcripts per million) of biosynthetic gene cluster transcripts associated with gene clusters annotated by antiSMASH 6 as ladderanes. The column color annotations from top to bottom correspond to water layer, sampling season, and sampling fraction. The row color annotations are color-coded to differentiate MAG fraction preference (DESeq2; $P < 0.05$; False Discovery Rate (FDR) = 5%) as well as the phylum the MAG expressing the transcript is affiliated with. Each individual row is the aggregated TPM of a ladderane biosynthetic cluster. Source data are provided as a Source Data file.
