# Supplementary material for: Diverse secondary metabolites are expressed in particle-associated and free-living microorganisms of the permanently anoxic Cariaco Basin
Source: Nat Commun. 2023 Feb 6;14:656. doi: 10.1038/s41467-023-36026-w (PMC9902471; doi:10.1038/s41467-023-36026-w)
Supplement: Supplementary file 3 — Description of Additional Supplementary Files [file 41467_2023_36026_MOESM3_ESM.pdf]

Description of Additional Supplementary Files:

Supplementary Data 1: Taxonomic classification of the 565 Cariaco MAGs, as well as additional related information including completeness and contamination estimates. Column 1 lists MAG IDs, column 2 lists taxonomic domain, column 3 denotes phylum, column 4 the class, column 5 the taxonomic order, column 6 the family, column 7 lists the genus, column 8 the species, column 9 lists the genome length (in megabases), column 10 lists the longest contig in the genome (in kilobases), column 11 lists the number of contigs in the genome, column 12 lists the GC content, column 13 denotes the maker lineage, column 14 the estimated genome completeness, column 15 the estimated genome contamination, column 16 the strain heterogeneity, column 17 the closest reference genome placement, column 18 lists the average nucleotide identity with between the genome and closest reference genome, column 19 is the taxonomic classification method, column 20 lists any notes from the GTDB-Tk classifier, column 21 lists the amino acid percent, column 21 lists the translation table used, and column 22 lists the relative evolutionary divergence value.

Supplementary Data 2: Relative abundance estimates generated using CoverM to map quality-filtered reads from each metagenomic sample to all Cariaco MAGs (the taxonomic classification of each MAG is also included). Each row in the metagenomic sample columns corresponds to the percent of total reads that mapped to a MAG; the first row in these columns corresponds the percent of reads from the metagenome that did not map to any of the MAGs. Column 1 lists MAG IDs, column 2 the taxonomic domain, column 3 the phylum, column 4 the class, column 5 the order, column 6 the family, column 7 the genus, column 8 the species, and columns 9-55 correspond to metagenomic samples and list the relative abundances of MAGs in each sample.

Supplementary Data 3: A summary of phyla of MAGs recovered from this study of Cariaco Basin that have no known genomic representatives as of yet from the Black Sea. Column 1 lists the taxonomic phylum.

Supplementary Data 4: A descriptive table of all the antiSMASH biosynthetic gene cluster annotations recovered from the Cariaco MAGs. Column 1 lists the MAG IDs, column 2 lists the taxonomic phylum, column 3 lists the biosynthetic gene cluster class, column 4 denotes which contig the cluster is on, column 5 lists the size in base pairs of the cluster, columns 6 and 7 list the start and end positions of the cluster in the contig (in base pairs), column 8 denotes whether one or more ends of the cluster overlap with a contig edge, column 9 denotes whether the cluster is part of a hybrid cluster, column 10 lists the location (in base pairs) of the biosynthetic cluster core, column 11 lists the detection rule used to classify the biosynthetic cluster.

Supplementary Data 5: Identification of MAGs and annotation of gene clusters that contain putative antibiotic resistance genes identified by ARTS. Column 1 lists MAG IDs, column 2 lists BGC identifications, column 3 lists cluster class, column 4 lists contig number in the MAG, column 5 the

sequence ID, column 6 the sequence location in the genome, column 7 the type of HMM resistance model, column 8 HMM profile, column 9 the description of the protein domain.

Supplementary Data 6: A metadata table summarizing the geochemical measurements and sampling information for each of the metagenomes and metatranscriptomes that were collected for this study. Column 1 denotes the metagenome name as listed on NCBI, column 2 the metatranscriptome name as listed on NCBI, column 3 the depth the same was taken from, column 4 the size fraction of the sample, column 5 the time point, column 6 the sample replicate, column 7 the name the same was referred to as in this study, column 8 lists the sample type, column 9 lists the year samples were taken, column 10 the redox regime where the sample was recovered from, column 11 the oxygen concentration (µM), column 12 the nitrate concentration (µM), and column 13 lists the sulfide concentration (µM).
