# Supplementary material for: Diverse secondary metabolites are expressed in particle-associated and free-living microorganisms of the permanently anoxic Cariaco Basin
Source: Nat Commun. 2023 Feb 6;14:656. doi: 10.1038/s41467-023-36026-w (PMC9902471; doi:10.1038/s41467-023-36026-w)
Supplement: Supplementary file 5 — Reporting Summary [file 41467_2023_36026_MOESM5_ESM.pdf]

nature portfolio

Corresponding author(s): Maria Pachiadaki, Virginia Edgcomb

Last updated by author(s): Jan 5, 2023

Reporting Summary

Nature Portfolio wishes to improve the reproducibility of the work that we publish. This form provides structure for consistency and transparency in reporting. For further information on Nature Portfolio policies, see our Editorial Policies and the Editorial Policy Checklist.

Statistics

For all statistical analyses, confirm that the following items are present in the figure legend, table legend, main text, or Methods section.

n/a	Confirmed	
☐	☒	The exact sample size (*n*) for each experimental group/condition, given as a discrete number and unit of measurement
☒	☐	A statement on whether measurements were taken from distinct samples or whether the same sample was measured repeatedly
☐	☒	The statistical test(s) used AND whether they are one- or two-sided<br>*Only common tests should be described solely by name; describe more complex techniques in the Methods section.*
☒	☐	A description of all covariates tested
☒	☐	A description of any assumptions or corrections, such as tests of normality and adjustment for multiple comparisons
☒	☐	A full description of the statistical parameters including central tendency (e.g. means) or other basic estimates (e.g. regression coefficient) AND variation (e.g. standard deviation) or associated estimates of uncertainty (e.g. confidence intervals)
☐	☒	For null hypothesis testing, the test statistic (e.g. *F*, *t*, *r*) with confidence intervals, effect sizes, degrees of freedom and *P* value noted<br>*Give P values as exact values whenever suitable.*
☒	☐	For Bayesian analysis, information on the choice of priors and Markov chain Monte Carlo settings
☒	☐	For hierarchical and complex designs, identification of the appropriate level for tests and full reporting of outcomes
☒	☐	Estimates of effect sizes (e.g. Cohen's *d*, Pearson's *r*), indicating how they were calculated

Our web collection on statistics for biologists contains articles on many of the points above.

Software and code

Policy information about availability of computer code

Data collection	No software was used.
Data analysis	Custom command line scripts and R scripts used in data analysis for this project are available in the following GitHub repository: https://github.com/d-mcgrath/cariaco_basin. The following open-source softwares were used: DESeq2 1.34.0, CoverM 0.6.1, GUNC 1.0.5, MetaBat2 2.12.1, GTDB-Tk 2.1.1, SPAdes 3.11.1 , CheckM 1.0.1161, antiSMASH 5.0, antiSMASH 6.0, Anvi'o 4, Mummer 3.23, Bowtie2 2.5.0, BWA<br>2.0, Trimmomatic 0.39, Samtools 1.16.1, BiG-SCAPE 1.1.4, Prodigal 2.6.3, InterProScan 581, Prokka 1.14.6, ARTS 2.0, Minimap2 2.24-r1122, umap 0.2.9.0, ggplot2 3.3.6, tidyverse 1.3.2, dbscan 1.1-11.

For manuscripts utilizing custom algorithms or software that are central to the research but not yet described in published literature, software must be made available to editors and reviewers. We strongly encourage code deposition in a community repository (e.g. GitHub). See the Nature Portfolio guidelines for submitting code & software for further information.

Data

Policy information about availability of data

All manuscripts must include a data availability statement. This statement should provide the following information, where applicable:

- Accession codes, unique identifiers, or web links for publicly available datasets
- A description of any restrictions on data availability
- For clinical datasets or third party data, please ensure that the statement adheres to our policy

The metatranscriptome and metagenome data generated in this study have been deposited in the NCBI database under accession code PRJNA326482 [https://www.ncbi.nlm.nih.gov/bioproject/?term=PRJNA326482]. The processed metagenome-assembled genomes (in FASTA format) and biosynthetic gene cluster files (in ZIP format) are available at OSF (https://osf.io/usm8r/). The biogeochemistry data from the CARIACO Basin Time Series Station for May to November 2014 are available through the Biological and Chemical Oceanography Data Management Office (BCO-DMO) at the Woods Hole Oceanographic Institution (https://www.bco-dmo.org/dataset/652313/data). The MIBiG 2.0 database is publicly available (https://mibig.secondarymetabolites.org/). Source data are provided with this paper.

Human research participants

Policy information about studies involving human research participants and Sex and Gender in Research.

Reporting on sex and gender	N/A
Population characteristics	N/A
Recruitment	N/A
Ethics oversight	N/A

Note that full information on the approval of the study protocol must also be provided in the manuscript.

Field-specific reporting

Please select the one below that is the best fit for your research. If you are not sure, read the appropriate sections before making your selection.

☐ Life sciences ☐ Behavioural & social sciences ☒ Ecological, evolutionary & environmental sciences

For a reference copy of the document with all sections, see nature.com/documents/nr-reporting-summary-flat.pdf

Ecological, evolutionary & environmental sciences study design

All studies must disclose on these points even when the disclosure is negative.

Study description	We mined metagenomes and performed differential gene expression analyses to show that redox potential and microbial lifestyles (particle-associated vs. free-living) influence the composition and production of secondary metabolites in the stratified water column of the Cariaco Basin, Venezuela.
Research sample	The research samples for both metagenomic and metatranscriptomic analyses were water samples that we sequentially filtered on board using two filter types (see below, and also "Sampling Strategy"). This allowed us to capture different fractions of the microbial community inhabiting different depths along the water column of the Cariaco Basin. The water samples were gravity-filtered through EMD Millipore 2.7 µm glass fiber membranes 47mm diameter to obtain the particle-associated fraction, and then through 0.2 µm Sterivex filters to capture the free-living fraction. All filters were stored frozen at -20 °C in the field and then at -80 °C in the laboratory until extraction (see also section "Data Collection").
Sampling strategy	Water samples were collected with Niskin bottles at different depths along the water column of Cariaco (103-900 m). These water depths were chosen based on the detected O2 concentrations that were measured in situ using Conductivity, Temperature, Depth (CTD) sensors mounted on the rosette holding the Niskin bottles. Our study focused in three water horizons that reflect the oxycline (103-237m; drop of oxygen from > 80uM down to 0.4uM), shallow anoxic (247-267; anoxic and H2S < 3uM), and euxinic (900m; anoxic and H2S > 15uM) conditions in Cariaco Basin. Water samples for DNA extractions dedicated to metagenome analyses, were collected with gravity-filtration sequentially through EMD Millipore 2.7 µm glass fiber membranes 47mm diameter (captures the particle-associated fraction of the microbial community), and then through 0.2 µm Sterivex filters (captures the free-living fraction of the microbial community) for metagenome analyses. Water samples for RNA extraction dedicated to metatranscriptome analyses were sequentially filtered through EMD Millipore 2.7 µm glass fiber filters (particle-associated fraction) and then through 0.2 µm Millipore Express polysulfone membranes (free-living fraction). All samples were stored at -20oC and temperatures below until further processing. Statistical methods to predetermine sample size was not performed. The volumes of water sampled (see below) were chosen according to previous literature which indicates adequate sampling sizes and filtering times that ensure integrity of the genetic material during in situ filtering. DNA is a stable molecule, which enables filtering of larger volumes of seawater, and an easier handling of samples until extraction. RNA degrades rapidly, and this requires minimizing the time of in situ filtering and the sample handling to avoid fast degradation.

Data collection	Water samples for metagenomic analyses were collected from 6 depths during two cruises in May 2014 (7-12 May 2014) and in November 2014 (5-10 November 2014) using Niskin bottles. 8-10 L water samples for metagenomic analysis were gravity-filtered sequentially through EMD Millipore 2.7 µm glass fiber membranes 47mm diameter (PA fraction), and then through 0.2 µm Sterivex filters (FL fraction) and stored frozen at -20oc in the field and then -80oc in the laboratory until extraction. Water samples were also collected and preserved in situ for isolation of RNA and construction of metatranscriptome libraries from depths selected to capture anoxic and sulfidic water layers. RNA sample collections were conducted with a "Microbial Sampler - Submersible Incubation Device" (MS-SID). Water (2 L) was sequentially filtered through EMD Millipore 2.7 µm glass fiber filters and then through 0.2 µm Millipore Express polysulfone membranes at depth. The filters were preserved immediately in situ with RNAlater®. Upon MS-SID retrieval, preserved filters were transferred to cryovials with additional RNAlater and stored frozen at -20oc in the field and then -80oc in the laboratory until extraction. Data on Conductivity, Temperature, Depth (CTD) were automatically collected with CTD sensors that were mounted on the rosette holding the Niskin bottles. MS-SID instrument was also mounted on the rosette. MS-SID Sensors were automatically collecting data for High Range Conductivity, Temperature, Density (Neil Brown Ocean Sensors, Incorporation, Falmouth, Massachusetts (MA), United States of America (USA)), Oxygen, (Aanderaa oxygen optode; Aanderaa Data Instruments Incorporation, Attleboro, MA, USA), Turbidity (2 NTURTD0-124 turbidity sensors; WET Labs Incorporation, Philomath, Oregon, USA), real-time bidirectional communication, and electronic control. The MS-SID possesses a Synchronous Digital Subscriber Line (SDSL) data link that multiplexes digital signals from up to 5 sensors at once, and bi-directionally transmits signals to and from the instrument for triggering "adaptive sampling" operations via ordinary conducting hydrowire.
Timing and spatial scale	Water samples were collected during two sampling cruises that took place in Cariaco Basin between 7-12 May 2014, and 5-10 November 2014, respectively. Seasonal differences in primary productivity can also shape microbial communities and the genes they express. In Cariaco Basin, there is a seasonal upwelling season (after January and until April) where the availability of nutrients results in maximum primary production that is lower during the non-upwelling season (October-December) due to the lower vertical carbon flux. The selection of the data collection period aimed to capture these two representative seasonal changes in Cariaco Basin. One location was sampled the Cariaco Time Series station (10.58N, 64.78W).
Data exclusions	No data were excluded from this study.
Reproducibility	Multiple casts were conducted. The presented results were produced from duplicate experiments.
Randomization	Randomization was not applicable to the study. The experimental design of this study did not involve controlled trials.
Blinding	Blinding was not applicable to the study. The experimental design of this study did not involve controlled trials.

Did the study involve field work? ☒ Yes ☐ No

Field work, collection and transport

Field conditions	No rainfall occurred during the sampling period. Water temperature ranged from 17.1 to 19.4oC.
Location	The research expedition took place in Cariaco Basin, Venezouela (10.50° N, 64.67° W). Elevation above sea level is 16m and maximum water depth ~1400 m. Water depths collected during this study range from 103-900 m.
Access & import/export	No permits were required. Co-author Gordon Taylor was the Principal Investigator of the Time Series Station that was established research relationships with Venezuela since 1995.
Disturbance	No disturbance to Cariaco Basin was caused because of the study.

Reporting for specific materials, systems and methods

We require information from authors about some types of materials, experimental systems and methods used in many studies. Here, indicate whether each material, system or method listed is relevant to your study. If you are not sure if a list item applies to your research, read the appropriate section before selecting a response.

Materials & experimental systems

n/a	Involved in the study
☒	☐ Antibodies
☒	☐ Eukaryotic cell lines
☒	☐ Palaeontology and archaeology
☒	☐ Animals and other organisms
☒	☐ Clinical data
☒	☐ Dual use research of concern

Methods

n/a	Involved in the study
☒	☐ ChIP-seq
☒	☐ Flow cytometry
☒	☐ MRI-based neuroimaging
